# Transcriptome and Metabolome Integration Reveals the Impact of Fungal Elicitors on Triterpene Accumulation in *Sanghuangporus sanghuang*

**DOI:** 10.3390/jof9060604

**Published:** 2023-05-24

**Authors:** Linjiang Zhou, Yan Fu, Xinyuan Zhang, Tong Wang, Guangyuan Wang, Liwei Zhou, Hailong Yu, Xuemei Tian

**Affiliations:** 1 Shandong Provincial Key Laboratory of Applied Mycology, College of Life Sciences, Qingdao Agricultural University, Qingdao 266109, China; 2 State Key Laboratory of Mycology, Institute of Microbiology, Chinese Academy of Sciences, Beijing 100101, China; 3 Institute of Edible Fungi, Shanghai Academy of Agricultural Sciences, National Engineering Research Center of Edible Fungi, Shanghai 201403, China

**Keywords:** medicinal mushroom, metabolomics, *Sanghuangporus sanghuang*, transcriptomics, triterpenoid

## Abstract

*Sanghuangporus sanghuang* is a large wood-decaying mushroom highly valued in traditional Chinese medicine due to its medicinal properties, including hypoglycemic, antioxidant, antitumor, and antibacterial properties effects. Its key bioactive compounds include flavonoids and triterpenoids. Specific fungal genes can be selectively induced by fungal elicitors. To investigate the effect of fungal polysaccharides derived from *Perenniporia tenuis* mycelia on the metabolites of *S. sanghuang*, we conducted metabolic and transcriptional profiling with and without elicitor treatment (ET and WET, respectively). Correlation analysis showed significant differences in triterpenoid biosynthesis between the ET and WET groups. In addition, the structural genes associated with triterpenoids and their metabolites in both groups were verified using quantitative real-time polymerase chain reaction (qRT-PCR) and high-performance liquid chromatography–tandem mass spectrometry (HPLC–MS/MS). Through metabolite screening, three triterpenoids were identified: betulinol, betulinic acid, and 2-hydroxyoleanolic acid. Excitation treatment increased the level of betulinic acid by 2.62-fold and 2-hydroxyoleanolic acid by 114.67-fold compared to WET. The qRT-PCR results of the four genes expressed in secondary metabolic pathways, defense gene activation, and signal transduction showed significant variation between the ET and WET groups. Overall, our study suggests that the fungal elicitor induced the aggregation of pentacyclic triterpenoid secondary metabolites in *S. sanghuang*.

## 1. Introduction

*Sanghuangporus sanghuang* is a type of fungus that belongs to the phylum Basidiomycota, class Agaricomycetes, order Hymenochaetales, family Hymenochaetaceae, and genus *Sanghuangporus*. It has been known in China for over 2000 years [1] and was previously misidentified as *Inonotus linteus* or *Inonotus baumii*. It was later distinguished as a new species, *Inonotus sanghuang*, in 2012 [2] and retitled *S. sanghuang* in 2016 [3]. Numerous studies have shown that *S. sanghuang* exhibits various biological activities such as hypoglycemic [4], antioxidant [5,6], antitumor, antibacterial [7], antidiabetic [8], and anti-inflammatory effects [9]. Triterpenoids are the main active components of *S. sanghuang* [5]. In addition, the anti-inflammatory role of *S. sanghuang* contributes to the alleviation of pulmonary symptoms induced by SARS-CoV-2, the pathogen responsible for the COVID-19 pandemic [6]. Therefore, *S. sanghuang* may help counter the effects of the new virus. In 1972, Keen defined an elicitor as a substance that stimulates the production and accumulation of biologically active components in plant cells [10]. Recent studies have expanded the definition of elicitor to include signaling molecules that induce defense responses, such as fungal polysaccharides, peptides, fermentation broth, and fungal secretions [11].

The natural yield of secondary metabolites synthesized by *S. sanghuang* is low and diverse. Therefore, it is important to explore methods for synthesizing more of the desired target metabolites. In recent years, the use of fungal elicitors to induce rapid and massive synthesis of target secondary metabolites in plant culture cells has become widespread. Liang et al. reported the induction of biosynthesis of terpenoid indole alkaloids in cambial meristematic cells of Catharanthus roseus using *Aspergillus flavus* fungal elicitor [12]. Li et al. selected three fungal strains (*Aspergillus niger*, *A. flavus*, and *Aspergillus oryzae*) as elicitors for mycelium or fermentation broth preparation to improve the yield of ginsenosides in adventitious root cultures of *Panax ginseng*. The results showed that ginsenosides production increased 3.52-fold with *A. niger* elicitor prepared from mycelium compared to the untreated group [13]. Prasad et al. researched the impact of fungal elicitors on the biomass and asparagine production of *Centella asiatica* multi-bud cultures in a dose- and time-dependent manner. The results demonstrate for the first time the potential application of *Trichoderma harzianum* CF in upregulating the asiaticoside biogenetic pathway in *C. asiatica* [14]. Therefore, the application of fungal elicitors to liquid fermentation cultures of medicinal fungi to increase secondary metabolite levels has a specific theoretical basis and practical significance.

As science and technology progress, more and more methods are being utilized for research, including proteomics, metabolomics, transcriptomics, genomics, and others. In order to develop high-throughput sequencing technology and systematic biology, multi-omics skills have become essential in biological sciences [15,16]. Young et al. reported that metabolite profiles and differentially expressed genes demonstrated significant synergistic effects on specific characteristic pathways based on the various growth stages of *Hypsizygus marmoreus*, and the flavor-related metabolites varied [17]. Ku et al. analyzed the transcriptome and metabolome of two cultivars of broccoli cultivated after jasmonic acid treatment. These biomarkers of metabolites and transcripts were used in effective marker-assisted breeding strategies to screen for insect-resistant cultivars [18]. Multiomics has become an invaluable analytical tool. The application of omics technology in edible fungi is becoming more and more extensive [19,20]. Therefore, this paper attempts to combine metabolomics and transcriptomics under conditions of elicitor treatment (ET) and without elicitor treatment (WET) to investigate the effects of fungal elicitors on the metabolites of *S. sanghuang*.

## 2. Materials and Methods

### 2.1. Preparation of Fungal Elicitor

After screening a large number of fungal elicitors [21], *Perenniporia tenuis* was selected as the elicitor resource strain in this study. The strain was cultured on a plate for 7 days and transferred to a corn starch liquid fermentation medium. The strain was then incubated at 28 °C and 150 rpm in a shaking culture for 7 days and subsequently filtered using a 200-mesh gauze. The mycelium was dried at 50 °C and then subjected to hot water extraction and alcohol precipitation [21]. An appropriate amount of alcoholic precipitate powder was dissolved in distilled water to obtain an aqueous solution of the polysaccharide elicitor.

### 2.2. Strains and Cultivation

The mycelium used in this study was from *S. sanghuang* CGMCC No. 21,068 (China General Microbiological Culture Collection Center, accession No. *S. sanghuang* 540), which was separated and cultured from a fruiting body on a Morus standing tree in a mountainous area of Anshun, Guizhou Province, China. The identification was performed by Professor Xuemei Tian using a combination of morphological and molecular biological methods, and the specimens were preserved in the Mycological Herbarium of Qingdao Agricultural University.

Before culturing with the added fungal polysaccharide elicitor (*P. tenuis*), fungal strains were inoculated on a liquid medium consisting of 20 g of wheat bran and 30 g of corn dissolved in distilled water and boiled for 25 min. Then, 30 g of glucose, 1 g of KH_2_PO_4_, 0.5 g of MgSO_4_._7_H_2_O, 4 g of yeast extract, and 3 g of peptone were added to the filtrate, and the mixture was incubated in the dark at 28 ± 1 °C for 7 days.

### 2.3. Fungal Elicitor Elicitation Test

Under sterile conditions, a liquid spawn of *S. sanghuang* was inoculated into an inactivated medium and incubated in a constant-temperature shaker at 28 °C and 150 rpm. After 1 day of incubation, the fungal polysaccharide elicitor was added to the shaker at a final concentration of 50 μg/mL (determined by anthrone–sulfuric acid colorimetry). 

Samples were taken in more than three bottles on the fourth, fifth, sixth, and seventh days of fermentation based on growth conditions to ensure viability. The test samples were quickly transferred to liquid nitrogen and stored in an ultralow-temperature refrigerator (−80 °C) for subsequent LC–MS/MS, RNA sequencing (RNA-seq), and qRT-PCR analysis. The mass specimens were also used for metabolome and transcriptome analysis, and full information was obtained based on three independent biological repetitions.

### 2.4. Sample Preparation and Analysis of Metabolism

#### 2.4.1. Specimen Preparation

The specimens were first lyophilized in a freeze-dryer (Scientz, Ningbo Scientz Biotechnology Co., Ltd., Scientz-100F, Ningbo, China) and then ground into powder form using a grinder (Retsch, Verder Shanghai Instruments and Equipment Co., Ltd., MM 400, Shanghai, China) at 30 Hz for 1.5 min.

One hundred milligrams of the powder were then dissolved in 1.2 mL of 70% methanol extract. The samples were vortexed for 30 s every 30 min for a total of six times and transferred to a refrigerator at 4 °C overnight. Finally, the samples were centrifuged at a rotation speed of 12,000 rpm for 10 min). The supernatant was aspirated, and the samples were filtered through a microporous membrane with a pore size of 0.22 μm and stored in an injection vial for ultra-performance liquid chromatography–mass spectrometry (UPLC–MS/MS) analysis. 

#### 2.4.2. Liquid Chromatography–Mass Spectrometry Analysis

The UPLC–MS/MS analysis was performed using an Agilent SB-C18 column (1.8 µm, 2.1 × 100 mm^2^) with ultrapure water (0.1% formic acid added) as phase A and acetonitrile (0.1% formic acid added) as phase B. The elution gradient was as follows: 5% phase B at 0.00 min, linear increase to 95% phase B at 9.00 min, maintained at 95% for 1 min (10.00–11.10 min), decreased to 5% phase B, and equilibrated with 5% for 14 min with a flow velocity of 0.35 mL/min, column oven at 40 °C, and infusion volume of 4 μL. The mass spectrometry was performed using linear ion trap (LIT) and triple quadrupole (QQQ) scans obtained on a Q TRAP triple quadrupole linear ion trap mass spectrometer. The AB4500 Q TRAP UPLC/MS/MS system was equipped with an electrospray ionization (ESI) turbo-ion atomized interface and controlled by Analyst 1.6.3 software (AB Sciex) to run in both positive and negative modes. The ESI source operating parameters were as follows: ion source, turbo spray; source temperature 550 °C; ion spray voltage (IS) 5500 V (positive-ion mode)/−4500 V (negative-ion mode); and ion source gas I (GSI), ion source gas II (GSII), and curtain gas set to 50, 60, and 25.0 psi, respectively. The collision-induced ionization parameters were set high. Instrument tuning and mass calibration were performed in QQQ and LIT modes using 10 and 100 μmol/L polypropylene glycol solutions. The QQQ scans were performed under the multiple reaction monitoring (MRM) mode with the inert gas (nitrogen) postulate medium. Further declustering potential (DP) and collision energy (CE) optimization were accomplished for individual MRM ion pairs, and a particular set of MRM ion pairs was checked in every stage based on the eluted metabolites in each stage.

#### 2.4.3. Metabolomic Data Analysis

The metabolites in the samples were qualitatively and quantitatively analyzed using mass spectrometry and a metabolic database. The metabolite detection multipeak plot obtained via MRM mode showed distinct metabolites in the sample corresponding to their mass spectrometry peaks. The signal intensity (cps) of the characteristic ion was obtained in the detector by screening the characteristic ion of each substance using a triple quadrupole. Offline mass spectrometry document MultiQuant software was used for integration and calibration of the peaks. The area of each peak represented the corresponding content, and the final total area of integrated information was exported and saved as the climax.

#### 2.4.4. RNA Extraction, cDNA Library Construction, and Sequencing

Total RNA from *S. sanghuang* mycelia stimulated for 4 days was extracted using an NEBNext^®^ UltraRNA Library Prep Kit (New England BioLabs, Ipswich, MA, USA) following the manufacturer’s instructions. The purity, concentration, and integrity of the RNA samples were evaluated using Nanodrop GX to ensure the use of high-quality specimens for transcriptome sequencing. mRNA was enriched using oligonucleotide (dT)-linked magnetic beads of mRNA Capture Beads (Novozymes, Bagsværd, Denmark) and then randomly fragmented using a fragment buffer. The cleaved RNA fragments served as templates for the first-strand cDNA synthesis via reverse transcription, followed by the formation of the second strand of cDNA using buffer, dNTPs, RNase H, and DNA polymerase. The double-stranded cDNA was purified using magnetic beads, end-to-end repaired, and poly-A tails were added before ligating to the sequencing connector. DNA clean beads (Novozymes) were used to select cDNAs of approximately 200 bp in length. PCR amplification was performed to enrich the cDNA template, and the library was finally sequenced using Illumina Hiseq 2000 (Illumina Co., Ltd., San Diego, CA, USA). Raw sequencing files were deposited to NCBI (SRA, https://www.ncbi.nlm.nih.gov/sra, accessed on 1 March 2022, Bioproject Accession: PRJNA779190, SRA Accession: SRR16883315 to SRR16883320).

#### 2.4.5. Transcriptome and Metabolomic Assembly and Annotation

To ensure high-quality and accurate data, the FASTA software was used to filter out unnecessary reads from the original information [22]. The chosen data was then converted into a transcriptome, and the unigene function was annotated using various databases, including non-redundant protein sequences from the NCBI (National Center for Biotechno, https://ftp.ncbi.nlm.nih.gov/blast/db/FASTA/, accessed on 12 December 2021), KEGG (Kyoto Encyclopedia of Genes and Genomes, https://www.genome.jp/kegg, accessed on 12 December 2021), COG (Clusters of Orthologous Groups of proteins, https://www.ncbi.nlm.nih.gov/COG/, accessed on 12 December 2021), GO (Gene Ontology, https://www.geneontology.org, accessed on 12 December 2021), Swiss-Prot (http://www.ebi.ac.uk/uniprot/, accessed on 12 December 2021), KOG (Karyotic Orthologous Groups, https://www.ncbi.nlm.nih.gov/KOG/, accessed on 12 December 2021), and Pfam (Protein family, https://www.Pfam.janelia.org, accessed on 12 December 2021).

#### 2.4.6. Expression of Triterpenoid Biosynthesis-Related Genes in *S. sanghuang* under Elicitor Treatment

Based on the studies conducted by Yang et al. [23] and Jeena et al. [24], three unigenes associated with secondary metabolic biosynthesis were selected and verified using qRT-PCR. The RNA was reverse transcribed into cDNA using a TUREscript 1st Stand cDNA Synthesis Kit (Aidlab, Qingdao, China). The qRT-PCR was conducted on a German Analytik Jena-qTOWER 2.2 real-time PCR system, using 2× SYBR Green Premix (Qingdao, China). Six biological repeats and three technical repeats were performed, and the names and sequences of the primers used in all experiments are provided in Table S2. For each reaction, a 10 µL volume was used, which contained SYBR Premix Ex Taq II (3 µL), forward primer (200 nM, 0.5 µL), reverse primer (200 nM, 0.5 µL), cDNA template (5 ng/µL, 1 µL), and ddH2O (3 µL). The qPCR parameters included an initial step of 95 °C for 3 min, followed by 40 cycles of amplification (95 °C for 10 s, 60 °C for 30 s). The melting curve was measured after holding at 60 °C to 95 °C for 4 s, with an increase of +1 °C/cycle, using the 18S gene as an internal control reference gene. Finally, the 2^−ΔΔCt^ method was used to calculate the gene expression [25].

### 2.5. Determination of Total Triterpene Content

The triterpene content was assessed by UV spectrophotometry [26]. The triterpene content was determined based on the amount of oleanolic acid equivalent (mg OAE/g) per gram of sample.

### 2.6. Detection of NO Content

The fresh mycelium sample was weighed and transferred to a pre-cooled mortar. Saline was added, and the sample was ground thoroughly until a homogenous solution was obtained. A 10% homogenate of the mycelium was prepared and centrifuged at 4 °C for 15 min at 2000 r/min. The supernatant was then discarded. The NO content of the sample was measured using a nitric oxide (NO) kit (Jiancheng Biotechnology Co., Ltd., Nanjing, China), following the guidelines specified in the operating manual.

### 2.7. Statistical Analysis

Entire transcriptome and metabolome samples were used as six biological replicates. PCA and Pearson correlation coefficient analysis were performed using software version 3.5.0, while heat map analysis was carried out using software version 1.0.12 (pheatmap). For OPLS-DA analysis, MetaboAnalyst version 1.0.1 was utilized with software parameters provided by Bioprofile Co., Ltd. (Minneapolis, MN, USA). Statistical evaluation was performed using an independent samples t-test between ET and WET.

## 3. Results

### 3.1. Identification of Metabolites

**Figure 1:**
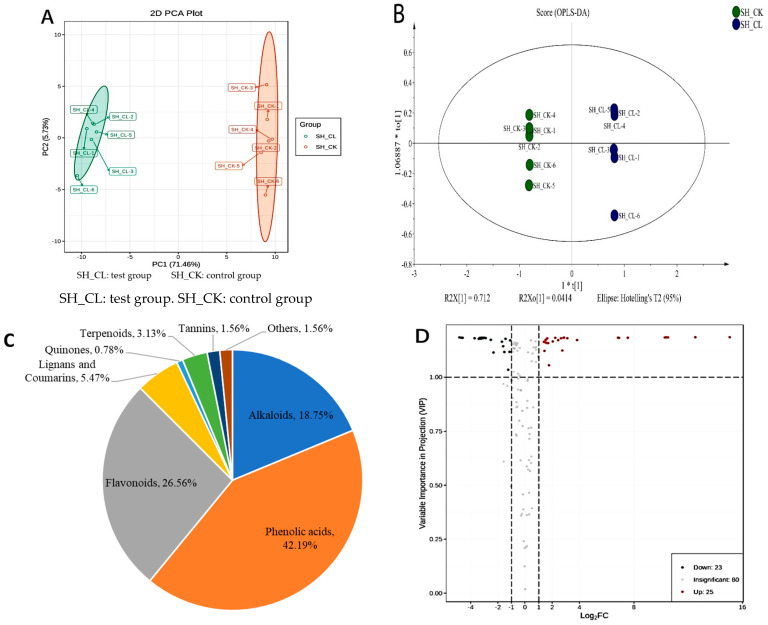
*S. sanghuang* samples from ET and WET groups were analyzed via UPLC−MS/MS to produce PCA and OPLS−DA score plots. (**A**) PCA score plots for ET (SH−CL) and WET (SH−CK) groups. The *x*-axis represents PC1, and the *y*−axis represents PC2. (**B**) t[1]: the first predicted principal; to[1]: the first orthogonal principal component. Plot of the OPLS−DA scores of presumptive annotated metabolites of ET and WET. The *x*−axis represents the scores of the main components in the orthogonal signal correction, and the difference between the groups can be determined from the orientation of the *x*−axis. The *y*−axis represents the fraction of orthogonal components in the orthogonal signal correction, and the difference between the groups can be determined from the orientation of the *y*−axis. (**C**) Metabolites classification statistical graphs. (**D**) Differential metabolite volcano map. In the graph, the green dots represent the downregulated differentially expressed metabolites; red dots represent the upregulated differentially expressed metabolites; and gray points represent the inspected metabolites, which were not significantly different.

The effect of the fungal elicitor on metabolites in the ET and WET groups was investigated through metabolome analysis. The results showed a significant overlap in the total ion chromatogram for metabolite detection, with consistent signals of the mass spectrum. The sample quality was homogeneous and stable, meeting the criteria for follow-up experiments. Principal component analysis (PCA) was performed on *S. sanghuang* samples from ET and WET groups, with PC1 and PC2 accounting for 71.46% and 5.73%, respectively, as shown in Figure 1A. An orthogonal partial least-squares discriminant analysis (OPLS-DA) of the metabolite profiles of *S. sanghuang* was also performed, with R2X, R2Y, and Q2 values of 0.766, 0.999, and 0.995, respectively (Figure 1B), indicating a stable and credible OPLS-DA model with Q2 > 0.9. The PCA and OPLS-DA score plots showed a clear separation between ET and WET, suggesting that the polysaccharide elicitors had an effect on metabolism in *S. sanghuang*.

A total of 128 metabolites were detected and identified in the ET and WET groups, mainly including flavonoids, phenolic acids, and alkaloids (Figure 1C). Based on the OPLS-DA results, multivariate analysis was initially performed on the metabolites of different samples using the variable importance in prediction (VIP) obtained from the OPLS-DA model. Univariate analysis was then combined with the *p*-value or fold change to further screen for differentially accumulated metabolites (DAMs). Significantly different metabolites were screened using a statistical difference multiplier value of fold change ≥ 2 or fold change ≤ 0.5, combined with a VIP value of ≥1 in the OPLS-DA model. Among these, 48 metabolites were identified as significantly differential secondary metabolites, with 25 upregulated and 23 downregulated (Table S2 and Figure 1D).

**Figure 2:**
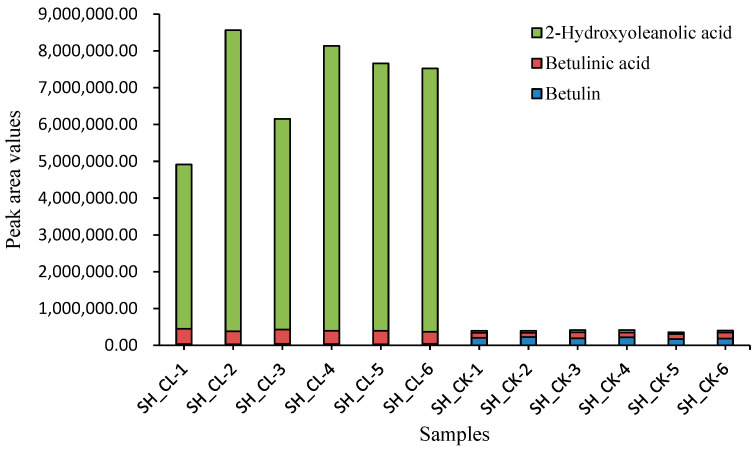
Filtering and validation of various metabolites revealed that betulinol was downregulated and betulinic acid and 2-hydroxyoleanolic acid were upregulated between ET and WET.

The results showed that the significantly different metabolites mainly included flavonoids, phenolic acids, and terpenoids (Table S2). Among them, dihydroxyoleanolic acid, a terpenoid, was significantly upregulated with a difference fold of 114.672, while betulinic acid showed a difference fold of 2.625, significantly higher than the control group (Additional File S2, Table S2). Three biphenylquinone and other terpenoid-quinone bioactivity-related triterpenoids were detected, including betulinol, which was downregulated, and betulinic acid and 2-hydroxyoleanolic acid, which were upregulated (Figure 2). These metabolites showed obvious differences between the ET and WET groups.

### 3.2. Variation in DAMs of Triterpenes

**Figure 3:**
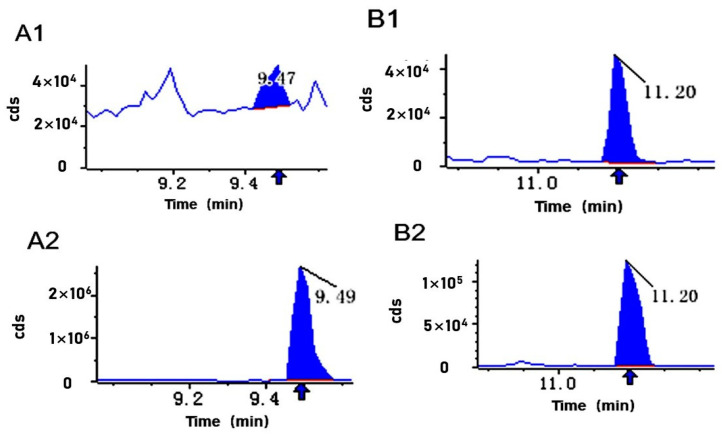
Integral calibration chart of quantitative metabolite analysis. The *x*-axis represents the maintenance time (min) for detecting metabolites, and the *y*-axis is the ion flow intensity (cps) for detecting metabolite ions. (**A1**) represents the content of 2-hydroxyoleanolic acid following WET. (**A2**) represents the content of 2-hydroxyoleanolic acid following ET treatment. (**B1**) represents the content of betulinic acid following WET treatment. (**B2**) represents the content of betulinic acid following ET.

To compare ET and WET, the differences between the two terpenoids, 2-hydroxyoleanolic acid and betulinic acid, in the two samples and to ensure accurate qualitative and quantitative analysis, their retention times and mass spectral peak areas were calibrated. Figure 3 shows the relative quantification of 2-hydroxyoleanolic acid and betulinic acid before and after exciton treatment, which is consistent with the metabolomics results, with significant upregulation of both terpenoids, indicating that the addition of elicitor in *S. sanghuang* had a significant effect on the terpenoids.

### 3.3. Filtering and Verification of Various Metabolites

**Figure 4:**
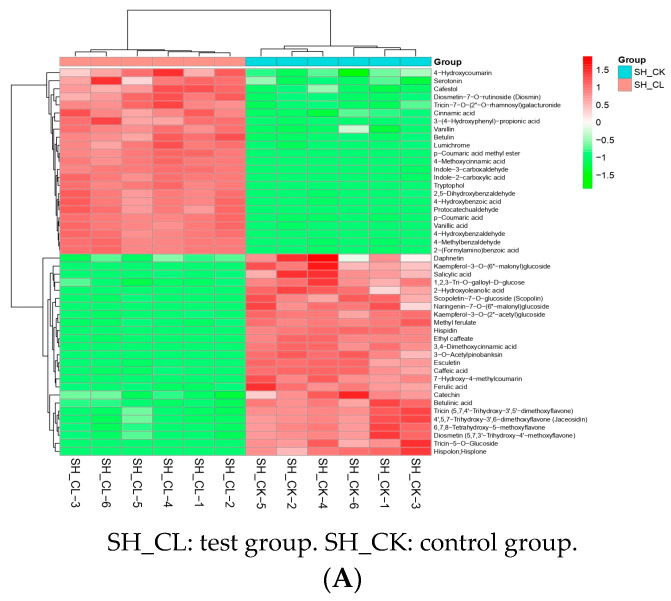

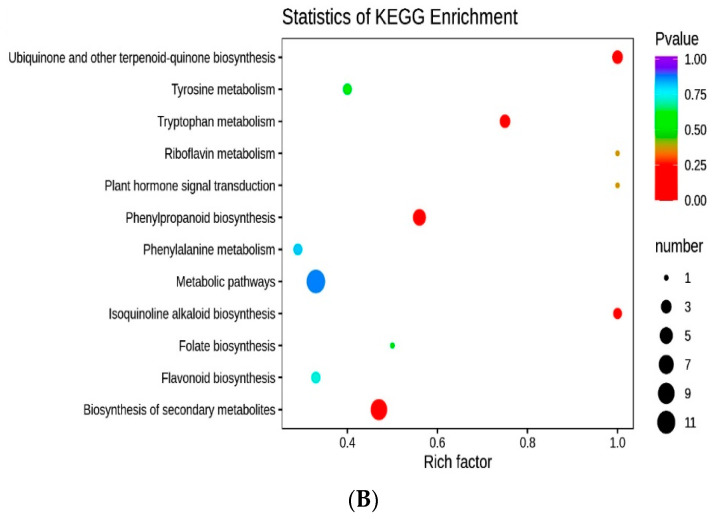
General overview of differential metabolites and pathway enrichment analysis of DAMs. (**A**) Heat map of differential metabolite clusters. The horizontal axis indicates the sample name; the vertical represents the differential metabolite information; and the clustering tree on the left of the figure indicates the differential metabolite clustering tree. Red represents the high content, and green indicates the low content. (**B**) Scatter plot of differential metabolites in KEGG enrichment analysis. The *p*-value approaching 0 depicts a more pronounced enrichment. The dimension of the points represents the corresponding number of the DAMs enriched.

To visualize the pattern of changes in metabolites, we normalized the metabolites with significant differences using the unit variance scaling method (UV) and generated heat maps using the R software package pheatmap, as shown in Figure 4A. The main classes of metabolites included phenolic acids, alkaloids, lignans and coumarins, flavonoids, and terpenoids. Among these, flavonoids (24.4%), terpenoids (8.8%), and lignans and coumarins (11%) showed significant upregulation compared to the control group. To gain further insight into the functions of DAMs and their related biological processes, we performed an enrichment analysis of DAMs using KEGG. The results indicated that DAMs were primarily enriched in pathways related to “ubiquinone and other terpenoid–quinone biosynthesis”, “biological synthesis of secondary metabolites”, “phenylpropanoid metabolism”, “tryptophan metabolism”, and “isoquinoline alkaloid biosynthesis” (Figure 4B).

### 3.4. Transcriptomic Analysis of S. sanghuang from ET and WET

The RNA-seq data obtained from ET and WET samples were evaluated, and the percentage of Q30 bases ranged from 94.69% to 95.66% (Table S2). The GC levels of WET and ET ranged from 52.52% to 52.63% and 52.34% to 52.56%, respectively (Table S2).

**Figure 5:**
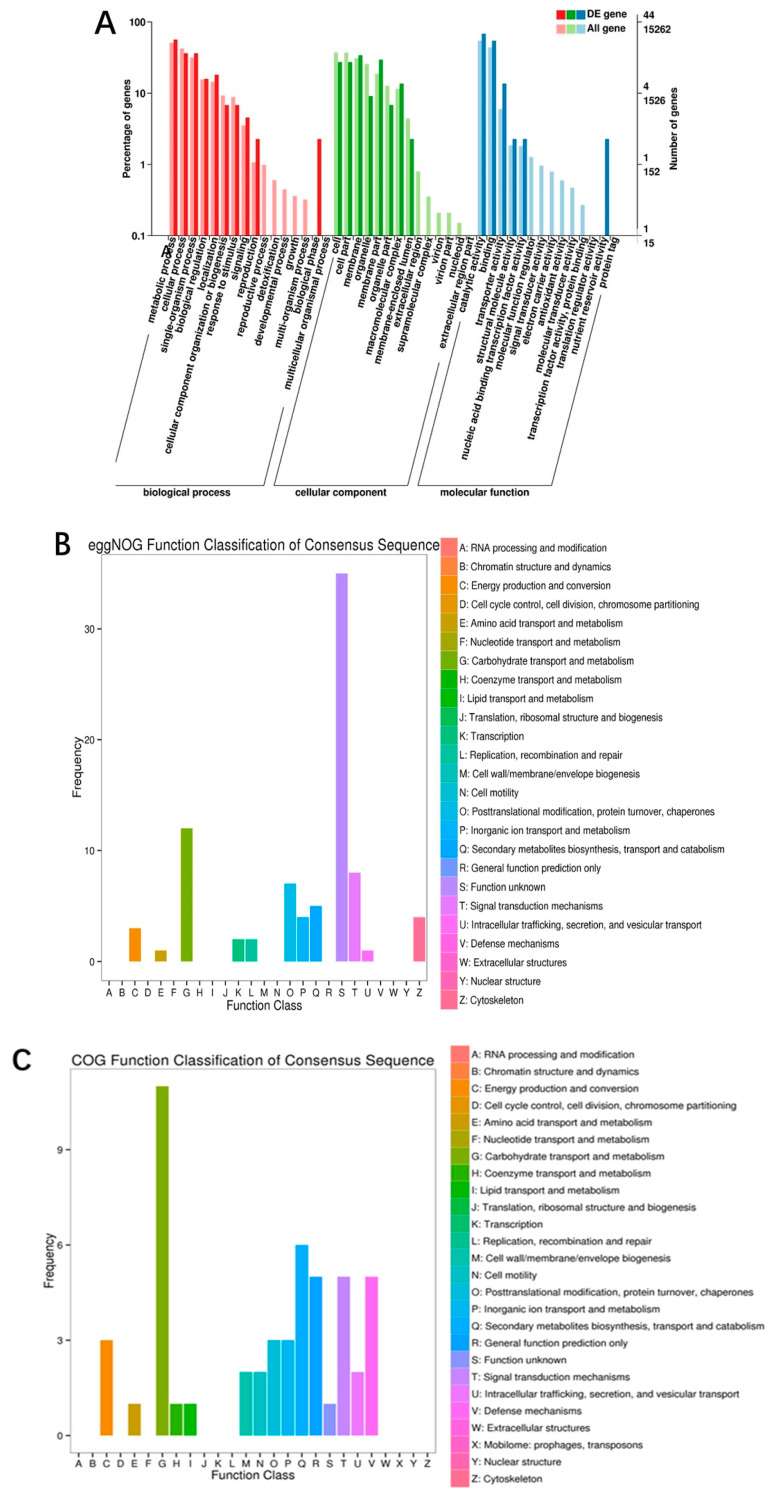
Classification of GO, COG, and egg NOG from the DETs between ET and WET. (**A**) GO annotation and enrichment of DETs. The horizontal coordinate is the GO grouping; the left side of the vertical coordinate represents the percentage of the number of transcripts, and the right side is the number of transcripts. (**B**) Egg NOG classification of DETs. The horizontal coordinate is the content of each category of egg NOG, and the vertical coordinate refers to the number of genes. (**C**) COG classification of DETs. The horizontal coordinate represents the level of each COG classification, and the vertical coordinate denotes the number of genes. The metabolic or physiological bias in the corresponding periods and environment is reflected by changes in the number of genes.

During the analysis, we identified 97 differentially expressed transcripts (DETs) between the control and stimulated groups, using a filter of fold change ≥ 2 and FDR < 0.01. Among these DETs, 57 were upregulated, and 40 were downregulated. To gain insight into the functions of these DETs in relevant biological processes, we performed Gene Ontology (GO) analysis. The GO analysis of DETs and their functional classification and enrichment analysis are shown in Figure 5A. A total of 112 DETs were identified in both the WET and ET groups. Among the biological processes, 40 DETs were significantly enriched in “metabolic processes”, “cellular processes”, “reproductive processes”, “signaling”, “multicellular biological processes”, “developmental processes”, “growth”, “stress response”, and “subcellular localization.” In terms of molecular function, 30 DETs were significantly enriched in “nucleic acid binding”, “transcription factor activity”, “catalytic activity”, “signal transduction”, “structural molecules”, “transport”, “binding”, “electron carriers”, “antioxidant activity”, “protein labeling”, “translation regulation”, and “nutrition.” Additionally, around 41 DETs were significantly enriched in “cells”, “cell membranes”, “macromolecular complex”, and “organelles” in cellular components.

To analyze the differential transcript protein annotation information, DETs were annotated to the egg NOG database (Figure 5B), and 47 functional groups were categorized. The DETs were mainly enriched in “carbohydrate transport and metabolism” (8, 17.0%), followed by “signal transduction mechanisms” (3, 6.4%) and “secondary metabolite biosynthesis, transport, and catabolism” (3, 6.4%). A large proportion of the remaining DETs were annotated to “replication, recombination, and repair” (2, 4.2%), “cytoskeleton” (2, 4.2%), “energy production and conversion” (2, 4.2%), and “inorganic ion transport and metabolism” (2, 4.2%). Only a small fraction of DETs were annotated to “transcription” (1, 2.1%). The transcriptional analysis revealed that the metabolic pathway of *S. sanghuang* was significantly affected by the transcriptional profile of ET. Figure 5C shows that the DETs were annotated to the COG database and categorized. The largest proportion of the 26 COG categories was accounted for by “carbohydrate transport and metabolism” (7, 26.9%), followed by “defense mechanisms” (3, 11.5%) and “secondary metabolite biosynthesis, transport, and catabolism” (3, 11.5%). A considerable portion of the remaining genes were annotated to “signal transduction mechanisms” (2, 7.7%), “intracellular transport”, “secretion and vesicular transport” (2, 7.7%), and “inorganic ion transport and metabolism” (2, 7.7%). Only a small proportion of DETs (less than 4%) were assigned to “cell motility” (1, 3.8%), “post-translational modifications, protein turnover, molecular chaperones” (1, 3.8%), “energy production and conversion” (1, 3.8%), and “coenzyme transport and metabolism” (1, 3.8%). Based on the results of annotation and enrichment analysis of differential metabolism of genes, the fungal elicitor effectively promoted the transcription of transport and metabolism of compound pathways, signal transduction pathways, defense mechanisms, and so on. They may play a significant role in terpene and sesquiterpene metabolic pathways and activation of triterpene synthesis in *S. sanghuang* (File S2, Table S2).

### 3.5. Verification of Differentially Expressed Transcripts (DETs) by qRT-PCR

**Figure 6:**
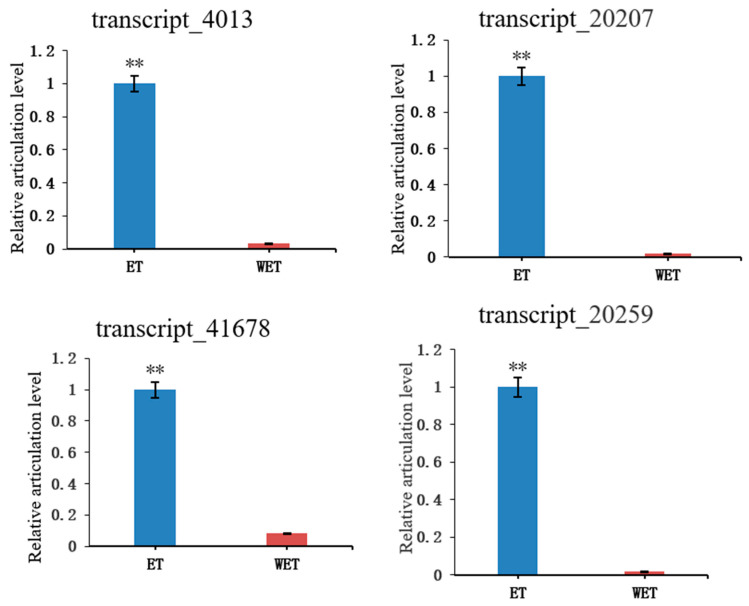
Expression levels of *S. sanghuang* under elicitor treatment. Transcript_×× represents the ID of the gene. The *y*-axis shows the relative articulation level of the genes by the 2^−∆∆Ct^ method. The *x*-axis represents the different handles. ** above the pillars show significant differences at *p* ≤ 0.01. Transcript_20259 and Transcript_41678: defense mechanisms (cytochrome P450 [*S. baumii*]); Transcript_20207: signaling pathways (hypothetical protein A7U60_g2886 [*S. baumii*]); Transcript_4013: sulfate anion transporter [*S. baumii*].

In the transcriptome data, genes related to signal transduction and defense mechanisms were identified. The results of each database annotation were as follows: “signal transduction” genes in GO functional annotation, “defense mechanism” (3, 11.5%) and “signal transduction mechanism” (2, 7.7%) genes in COG database annotation, and “signal transduction mechanism” (2, 7.7%) genes in the egg NOG database annotated with “signal transduction mechanism” (3, 6.4%) genes. To verify differences in transcript expression following elicitor treatment, four transcripts related to secondary metabolism, including defense mechanisms (Transcript_20259 and Transcript_41678: cytochrome P450 [*Sanghuangporus baumii*]) and signaling pathways (Transcript_20207: hypothetical protein A7U60_g2886 [*S. baumii*] and Transcript_4013: sulfate anion transporter [*S. baumii*]), were analyzed by qRT-PCR using designed primers. As shown in Figure 6, there was a highly significant difference between ET and WET, with ET facilitating DETs. The findings were consistent with the results of transcriptome analysis, which verified the authenticity of the transcriptome data (File S1, Figures S2–S5). 

### 3.6. Correlation between DAMs and DETs

**Figure 7:**
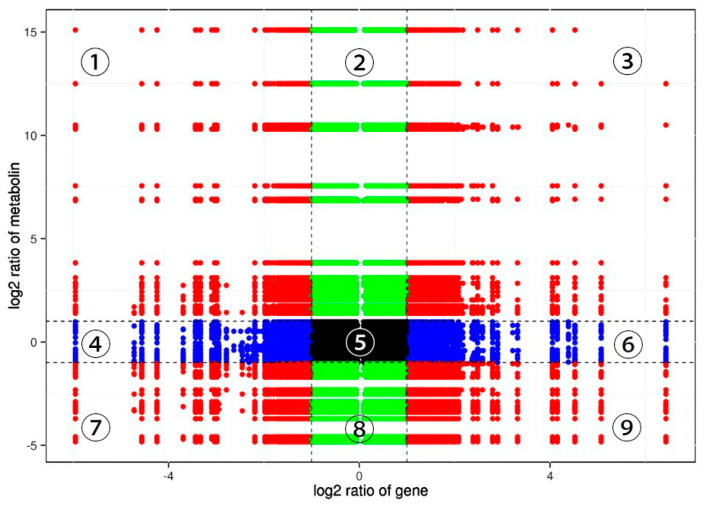
A nine−quadrant diagram representing the association between DAMs and DETs of *S. sanghuang* after elicitor treatment t. The *x*−axis represents the log ratio of differential genes. The *y*−axis represents the log ratio of differential metabolites; they are partitioned into ①−⑨ quadrants left to right and top to bottom with black dashed lines. The black dashed lines represent the different thresholds. Each dot represents a gene or metabolite, black dots represent the unchanged genes or metabolites; green dots represent DAMs with unchanged genes; blue dots represent differentially expressed genes with unchanged metabolites; red dots represent both differentially expressed genes and DAMs. Quadrant ⑤ represents unchanged genes and metabolites; quadrants ①, ②, and ④ represent metabolite expression abundance over genes; quadrants ③ and ⑦ indicate genes whose expression patterns are consistent with those of metabolites; and quadrants ⑥, ⑧, and ⑨ represent differentially expressed metabolites with lower expression abundance than differentially expressed genes.

Relevance analyses of DAMs and DETs were performed. The metabolites and the corresponding genes were screened based on the correlation coefficient (CC) > 0.80 and the *p*-value of correlation < 0.05. As shown in Figure 7, DAMs and differentially expressed genes shared a positive relationship in the third and seventh quadrants. We connected diverse gene clusters with differential triterpene metabolites in the third quadrant, indicating that after excitation, signal transduction mechanisms; biosynthesis, transport, and catabolism of secondary metabolites; and defense mechanisms are closely related to the production of pentacyclic triterpene metabolites 2-hydroxyoleanolic acid and betulinic acid. 

### 3.7. Effect of Mevalonate Pathways in ET Group

**Figure 8:**
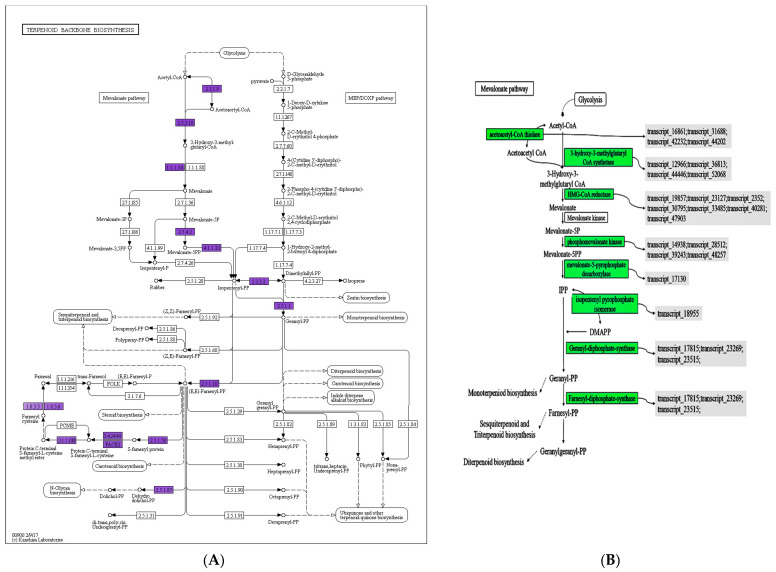
Effect of elicitor treatment on enzymes. (**A**) KEGG annotation; the purple in the diagram is the enzyme that is changed in the pathway. (**B**) Metabolic pathway, green denotes upregulated enzyme expression; gray denotes transcript number. Transcripts were annotated as those of a similar species, *Sanghuangporus baumii*. Transcript_16861; Transcript_31688; Transcript_42232; Transcript_44202: *Sanghuangporus baumii* acetyl-CoA acetyltransferase gene. Transcript_12966; Transcript_36813; Transcript_44446; Transcript_52068: *S. baumii* hydroxymethylglutaryl-CoA synthase mRNA. Transcript_19857; Transcript_23127; Transcript_2352; Transcript_30795; Transcript_33485; Transcript_40281; Transcript_47903: *S. baumii* 3-hydroxy-3-methylglutaryl-coenzyme A reductase (HMGR) mRNA. Transcript_14938; Transcript_28512; Transcript_39243; Transcript_48257: *S. baumii* phosphomevalonate kinase mRNA. Transcript_17130: *S. baumii* mevalonate pyrophosphate decarboxylase mRNA. Transcript_18955: *S. baumii* isopentenyl diphosphate isomerase mRNA. Transcript_17815; Transcript_23269; Transcript_23515: *S. baumii* farnesyl-diphosphate synthase mRNA.

The mevalonate pathway (MVA pathway) is a metabolic pathway for terpenoids, which produces isopentene pyrophosphate (IPP) in the cytoplasm using acetyl CoA as the primary donor. The transcriptome analysis showed that the ET treatment upregulated the enzymes involved in the MVA pathway and promoted the accumulation of terpenoids, as depicted in Figure 8. The KEGG annotation revealed that all enzymes in this pathway were affected, except MVK, with multiple enzymes being regulated by different transcripts. HMG-CoA reductase, a key rate-limiting enzyme of the MVA pathway, was annotated by seven genes at the transcriptional level. It catalyzes the synthesis of mevalonate from acetyl CoA, which, in turn, synthesizes IPP by further enzymatic action and generates dimethylallyl pyrophosphate (DMAPP), another precursor of terpenoids. Additionally, ET treatment promoted the synthesis of GPP and FPP, which are important precursors of terpenoids, thus positively affecting the synthesis of monoterpenes, diterpenes, and triterpenes.

Mevalonate kinase (MVK) is a rate-limiting enzyme in the MVA pathway, and its activity level plays a crucial role in the rate of terpenoid synthesis, affecting the yield of terpenoids significantly. However, the impact of fungal elicitor on MVK’s transcriptional expression was not evident in this experiment.

### 3.8. Detection of NO Content

**Figure 9:**
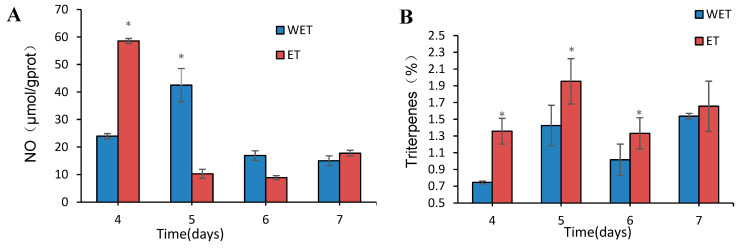
(**A**) The content of NO in *S. sanghuang* under elicitor treatment. (**B**) The content of total triterpenes in *S. sanghuang* under elicitor treatment. * above the pillars show significant differences.

Nitric oxide (NO) plays a vital role in the triterpene signaling pathway. To determine whether ET affects the signaling pathway of *S. sanghuang* and the authenticity of the transcriptome results, we measured the NO content (Figure 9A). On the fourth day of elicitor addition, the NO content was significantly higher than in the WET group. The results demonstrated that ET induced the production of the signaling molecule NO in *S. sanghuang*, thus validating the transcriptome data at the molecular level. 

### 3.9. Detection of Total Triterpene Content

To further verify the effect of fungal elicitor on the triterpene content of *S. sanghuang*, the total triterpene content was measured during the entire fermentation process. It was observed that after the addition of the fungal polysaccharide elicitor, the triterpene content of *S. sanghuang* showed an overall increase, followed by a decrease and then a subsequent increase. The maximum total triterpene accumulation was observed on day 5 of the fermentation process, as shown in Figure 9B. The highest total triterpene content was 1.95%, which is a 37.11% increase compared to that of the WET group. These results further confirm that the addition of the fungal elicitor effectively increases the yield of total triterpenes in *S. sanghuang*. 

## 4. Discussion

Triterpenoids are an important class of secondary metabolites composed of six isoprene units and have gained significant attention in recent years due to their pharmacological activity [27,28]. Fungal elicitors have been shown to promote liquid fermentation by fungi to obtain desired substances. For example, Cai et al. studied the impact of yeast extracts and specific polysaccharide elicitors on secondary metabolites, including anthocyanins and phenolic acids, in the suspension cultivation of *Vitis vinifera* [29]. Mercier et al. described the effects of two algal polysaccharide elicitors, laminarin and carrageenan, on defense reactions and signaling in tobacco plants [30]. In this study, a plant secondary metabolite database was constructed based on UPLC–MS/MS to identify 48 differential secondary metabolites, including phenolic acids, lignans and coumarins, flavonoids, and terpenoids, in polysaccharide elicitor-induced mycelium of *S. sanghuang*. Flavonoids (24.4%), terpenoids (8.8%), and coumarin and lignans (11%) showed significant upregulation. The fungal elicitor promoted the accumulation of triterpene and ketone metabolites in *S. sanghuang*. Among the triterpenoids, the levels of betulinic acid and 2-hydroxyoleanolic acid were 2.62-fold and 114.67-fold higher than in the control group, respectively. These compounds have been reported to have various pharmacological effects, such as antimalarial, anti-inflammatory [31,32], antibacterial [33], and antitumor properties [34,35].

An analysis of the full-length transcriptome sequencing of *S. sanghuang*, which generated 30,704 transcripts, was conducted to investigate the effect of fungal elicitor on metabolites. Using fold change ≥ 2 and FDR < 0.01 as criteria, 97 DETs were identified between the control and stimulated groups, of which 57 were upregulated and 40 were downregulated. The DETs were annotated into GO, KEGG, COG, and egg NOG databases and categorized. The results of gene annotation and enrichment analysis suggested that the fungal elicitor played a significant role in activating the secondary metabolic pathway and defense genes of *S. sanghuang*. Additionally, four stable genes related to secondary metabolism were identified based on the transcriptome sequencing results, and their differential expression was validated.

The results of the KEGG enrichment analysis revealed a significant enrichment of differential metabolites in the biosynthetic pathway of urea and other terpenoids-quinone, which is closely associated with the biosynthetic pathway of the terpene skeleton. Downregulation of 4-hydroxybenzoate in this pathway of urea and other terpenoids-quinone resulted in decreased consumption of polyisopentene diphosphate in the biosynthetic pathway of the terpene skeleton. This, in turn, led to a decrease in the production of branching acids, such as isobranchic acid and 1,4-dihydroxy-2-naphthoic acid, and a reduction in the consumption of polyisopentenyl diphosphate, which accumulates significantly in the biosynthetic pathway of the terpene skeleton. The decrease in the consumption of phytosubstituted monophosphate diammonium salts in the terpene skeleton biosynthetic pathway reduced the synthesis of 1,4-dioxo-2-naphthoic acid, causing its accumulation. Due to the low consumption, the accumulation in the biosynthetic pathway of the terpene skeleton led to the accumulation of geranylgeranyl pyrophosphate triammonium salt, which can be involved in other pathways that synthesize other terpenoids, such as the biosynthetic pathway of diterpenoids, the biosynthesis of indole diterpenoid alkaloids, and the biosynthesis of monoterpenoids. Based on these findings, it is possible that the accumulation and production of terpenoids are a result of the chain reactions mentioned above.

Currently, some studies have confirmed the role of NO signaling in promoting fungal metabolite accumulation in response to elicitors [36,37], but the investigation of the related mechanism using multi-omics combined analysis has not been reported. In this study, to investigate the factors affecting triterpene accumulation, we measured NO content. On the fourth day of addition, the NO content was significantly higher than that of the WET group. Transcriptome assays and indices simultaneously revealed that NO was involved in defense mechanisms and triterpene accumulation in *S. sanghuang*, with significant differences between WET and ET. This finding also suggests that the elicitor may have induced NO signaling and consequently promoted intracellular triterpene accumulation. 

The focus of this research was on the application of fungal elicitors in the liquid fermentation of *S. sanghuang* and their effect on secondary metabolism, studied through multi-omics analysis. The results indicated that fungal elicitors promote the accumulation of terpenoids, ketones, coumarins, and lignans, particularly ketones. However, the possible mechanisms behind these findings require further investigation. As medicinal mushrooms, the use of fungal elicitors in fungal liquid fermentation is critical for the study of triterpenes, the detection of fungal metabolites with medicinal properties, the preservation of existing natural resources, and the exploration of new products. The large-scale production of active ingredients, such as *S. sanghuang* triterpenes, is both of great interest to human health and has practical applications.

## Data Availability

All data presented in the study are included within the article and its supplementary files or have been deposited in NCBI with accession PRJNA779190.

## Supplementary Materials

The following supporting information can be downloaded at: https://www.mdpi.com/article/10.3390/jof9060604/s1.

## Abbreviations

CC	correlation coefficient
CE	collision energy
CMCs	cambial meristematic cells
COGs	clusters of orthologous groups of proteins
CUR	curtain gas
DAMs	differentially accumulated metabolites
DEGs	differentially expressed genes
DETs	differentially expressed transcripts
DP	de-clustering potential
ESI	electrospray ionization
ET	elicitor-treated
GO	gene ontology
GPI-APs	glycosylphosphatidylinositol-anchored proteins
GSI	ion source gas I
GSII	ion source gas I
HPLC–MS/MS	high-performance liquid chromatography–tandem mass spectrometry
IS	ion spray voltage
KEGG	Kyoto Encyclopedia of Genes and Genomes
KOG	Eukaryotic Orthologous Groups
LIT	linear ion trap
MRM	multiple reaction monitoring
NEB	NEBNext^®^ Ultra™ RNA library prep kit
NO	nitric oxide
NOS	nitric oxide synthase
Nr	non-redundant protein sequences
OPLS-DA	orthogonal partial least-squares discriminant analysis
PC1	principal component 1
PC2	principal component 2
PCA	principal component analysis
QQQ	triple quadrupole
QRT-PCR	quantitative real-time polymerase chain reaction
Q TRAP	triple quadrupole linear ion trap mass spectrometer
RNA-seq	RNA sequencing
*S. sanghuang*	*Sanghuangporus sanghuang*
SH-CL (ET)	elicitor-treated
SH-CK (WET)	without elicitor treatment
TIAs	terpenoid indole alkaloids
UPLC–MS/MS	ultra-performance liquid chromatography–mass spectrometry
UV	unit-variance scaling
VIP	variable importance in projection
WET	without elicitor treatment
