# Supplementary material for: Transcriptome and Metabolome Integration Reveals the Impact of Fungal Elicitors on Triterpene Accumulation in Sanghuangporus sanghuang
Source: J Fungi (Basel). 2023 May 24;9(6):604. doi: 10.3390/jof9060604 (PMC10299497; doi:10.3390/jof9060604)
Supplement: Supplementary file 1 [file jof-09-00604-s001.zip › Supplementary Materials1 Figure.pdf]

Applied Microbiology and Biotechnology

Supplementary Files

Combined Metabolome and Transcriptome Analyses of the Effects of Elicitors Treated on Triterpene Accumulation in *Sanghuangporus sanghuang*

Linjiang Zhou [1+] · Yan Fu[1+] · Xinyuan Zhang [1] · Tong Wang [1] · Guangyuan Wang [1] ·

Liwei Zhou [2] · Hailong Yu[3,*] · Xuemei Tian [1,*]

1 college of Life Sciences, Shandong Provincial Key Laboratory of Applied Mycology, Qingdao Agricultural University, Shandong Qingdao 266109, China; zhoulj24@163.com (L.J.Z.); xiaofu15662034968@163.com (Y.F.); 1172532789@qq.com (X.Y.Z.);
1173391395@qq.com (T.W.); gywang@qau.edu.cn (G.Y.W.)
2 State Key Laboratory of Mycology, Institute of Microbiology, Chinese Academy of Sciences, Beijing 100101, China; liwei_zhou1982@im.ac.cn (L.W.Z.)
3 Institute of Edible Fungi, Shanghai Academy of Agricultural Sciences, National Engineering Research Center of Edible Fungi, Shanghai, 201403, China. yuhailong@saas.sh.cn (H.L.Y.)
[+] There authors contributed equally to the this work.
* Corresponding author: txm@qau.edu.cn (X.M.T.), yuhailong@saas.sh.cn (H.L.Y.)

Figure Legends

Fig S1. 18S internal reference gene. The left graph shows the amplification curve， and the right graph shows the dissolution curve.
Fig S2. Transcripts 20207. The left graph shows the amplification curve， and the right graph shows the dissolution curve.
Fig S3. Transcripts 20259. The left graph shows the amplification curve， and the right graph shows the dissolution curve.
Fig S4. Transcripts 41678. The left graph shows the amplification curve， and the right graph shows the dissolution curve.
Fig S5. Transcripts 4013. The left graph shows the amplification curve， and the right graph shows the dissolution curve.

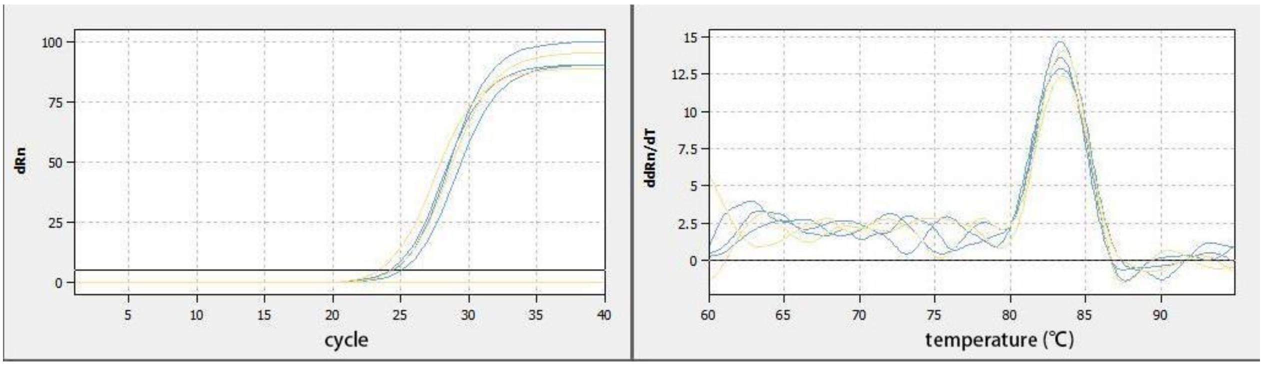

Fig S1. 18S internal reference gene

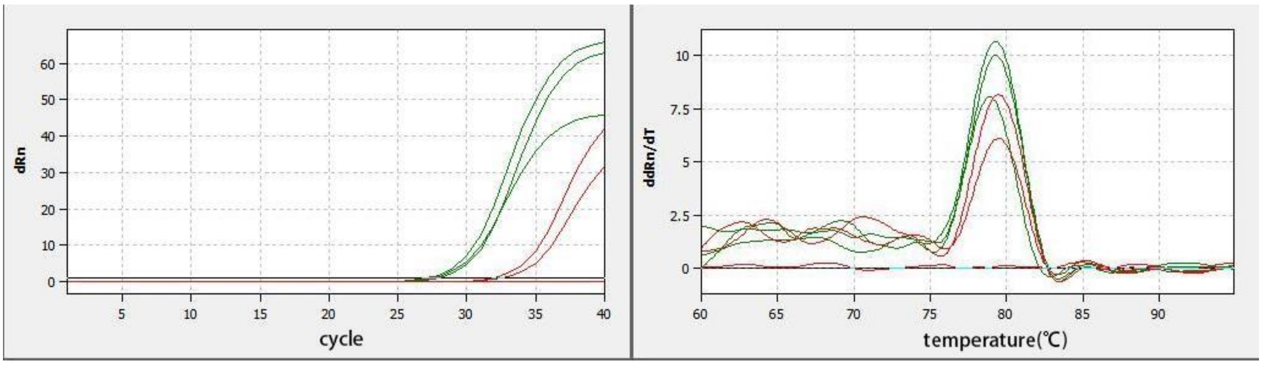

Fig S2. Transcripts 20207

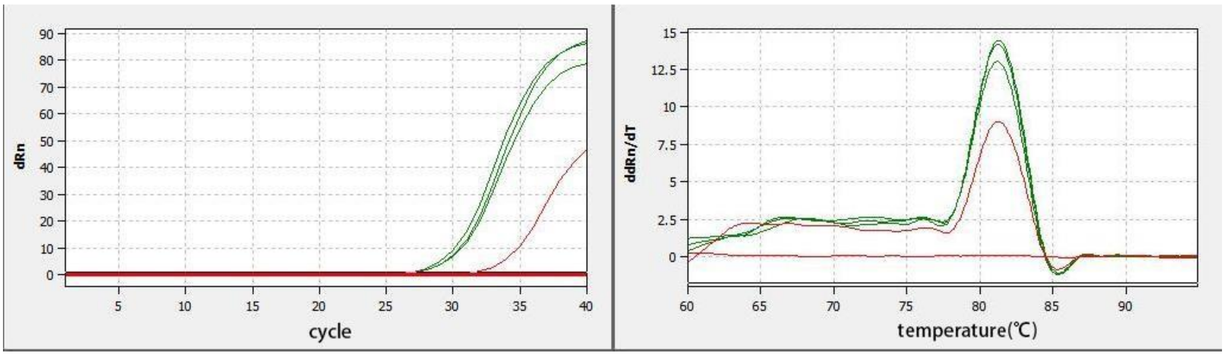

Fig S3. Transcripts 20259

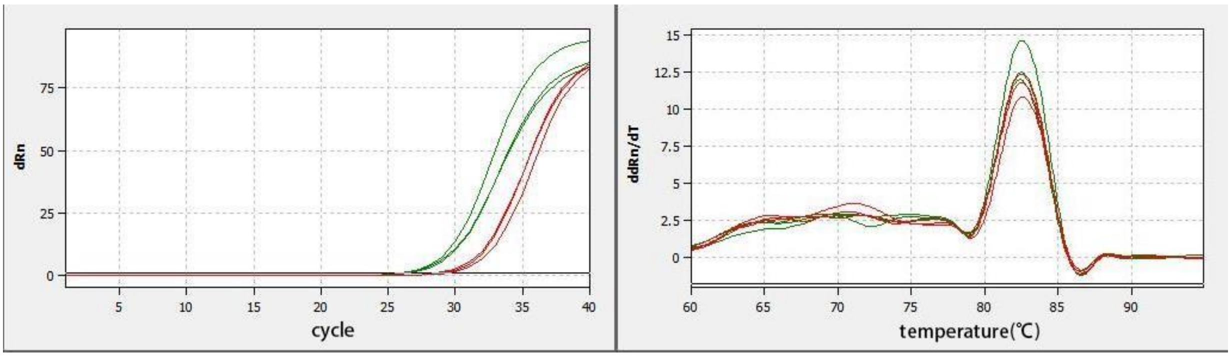

Fig S4. Transcripts 41678

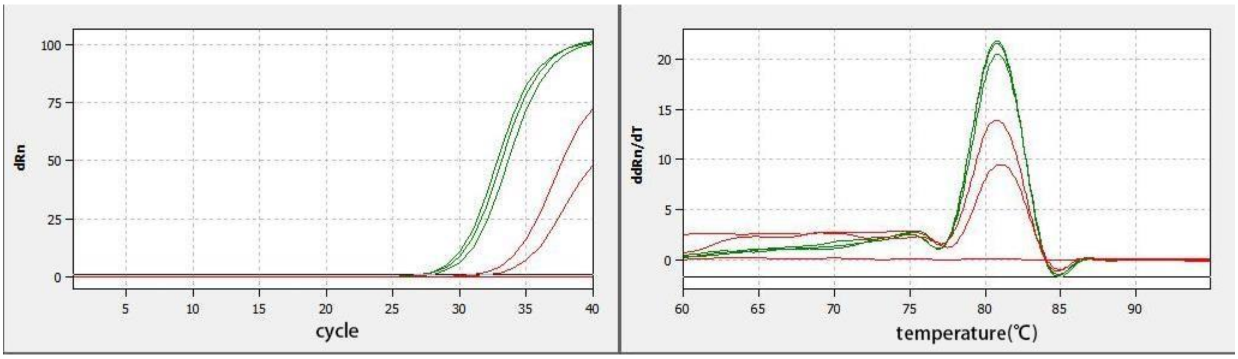

Fig S5. Transcripts 4013
